# Bacterial Diversity Dynamics Associated with Different Diets and Different Primer Pairs in the Rumen of Kankrej Cattle

**DOI:** 10.1371/journal.pone.0111710

**Published:** 2014-11-03

**Authors:** Dipti W. Pitta, Nidhi Parmar, Amrut K. Patel, Nagaraju Indugu, Sanjay Kumar, Karsanbhai B. Prajapathi, Anand B. Patel, Bhaskar Reddy, Chaitanya Joshi

**Affiliations:** 1 Center for Animal Health and Productivity, School of Veterinary Medicine, University of Pennsylvania, Philadelphia, Pennsylvania, United States of America; 2 Ome Research Facility, Department of Animal Biotechnology, Anand Agricultural University, Anand, Gujarat, India; 3 Livestock Production and Management Department, College of Veterinary Science and Animal Husbandry, Sardar Krushi Nagar Dantiwada Agricultural University, Sardar Krushi Nagar, Gujarat, India; International Atomic Energy Agency, Austria

## Abstract

The ruminal microbiome in herbivores plays a dominant role in the digestion of lignocellulose and has potential to improve animal productivity. Kankrej cattle, a popular native breed of the Indian subcontinent, were used to investigate the effect of different dietary treatments on the bacterial diversity in ruminal fractions using different primer pairs. Two groups of four cows were assigned to two primary diets of either dry or green forages. Each group was fed one of three dietary treatments for six weeks each. Dietary treatments were; K1 (50% dry/green roughage: 50% concentrate), K2 (75% dry/green roughage: 25% concentrate) and K3 (100% dry/green roughage). Rumen samples were collected using stomach tube at the end of each dietary period and separated into solid and liquid fractions. The DNA was extracted and amplified for V1–V3, V4–V5 and V6–V8 hypervariable regions using P1, P2 and P3 primer pairs, sequenced on a 454 Roche platform and analyzed using QIIME. Community compositions and the abundance of most bacterial lineages were driven by interactions between primer pair, dietary treatment and fraction. The most abundant bacterial phyla identified were *Bacteroidetes* and *Firmicutes* however, the abundance of these phyla varied between different primer pairs; in each primer pair the abundance was dependent on the dietary treatment and fraction. The abundance of *Bacteroidetes* in cattle receiving K1 treatment indicate their diverse functional capabilities in the digestion of both carbohydrate and protein while the predominance of *Firmicutes* in the K2 and K3 treatments signifies their metabolic role in fibre digestion. It is apparent that both liquid and solid fractions had distinct bacterial community patterns (P<0.001) congruent to changes in the dietary treatments. It can be concluded that the P1 primer pair flanking the V1–V3 hyper-variable region provided greater species richness and diversity of bacterial populations in the rumen of Kankrej cattle.

## Introduction

The bovine populations of the Indian subcontinent represent a diverse genetic resource formed through various natural selective pressures such as varying supplies of nutrients, climatic conditions, and within species competition. Further, local environment and economic traits continues this selection process which leads to shaping entirely new species [1], [2].

Based on phenotypic characterization, the National Bureau of Animal Genetic Resources reported 30 cattle breeds in India. Over millions of years ruminants and rumen microbiota have co-evolved and thus the rumen contains a complex and diverse bionetwork of bacteria, fungi and protozoa that facilitate fibre digestion. Unlike developed countries, domestic ruminants in developing and under-developed countries are often fed an abundance of fibre and little protein supplement (concentrate mix). When ruminants are fed fibre-rich rations the microbial ecology is altered. Since bacteria play an important role in all facets of rumen fermentation it is important to understand the rumen microbial ecology in domesticated ruminants that are maintained on local forages.

The breed Kankrej originated from Zebu cattle, native to the North Western part of India and is known for its dual (milk and draught) purpose and resilience to tropical weather conditions [3]. Kankrej cattle are native to the state of Gujarat and are held in high prestige there, being known to thrive on locally available forages with an average milk production of 6–10 L per day with 5% fat (unpublished data). As the composition of the rumen microbiome is primarily driven by diet [4] and the fact that Kankrej can utilize locally available feed resources efficiently for milk production, we were interested to determine diet-induced shifts in the rumen microbiome of Kankrej cattle.

In the recent past, next generation sequencing technology offered the most cost-effective platform to characterize community microbial populations at much greater resolution. Recently, we explored diversity in the metabolically active bacterial communities of water buffalo recovered by different primer pairs and investigated diet-induced shifts in the bacterial community compositions when water buffaloes were fed different proportions of forage and concentrate [5]. In this study, we used 454 Roche sequencing technology to investigate dynamics in the rumen microbiome of Kankrej cattle fed different roughages sources (dry and green) supplemented with a commercially available concentrate mixture.

## Materials and Methods

All animal management and research procedures were conducted under animal use protocols approved by the University Animal Ethics Committee (Permit number: AAU/GVC/CPCSEA-IAEC/108/2013), Anand Agricultural University (AAU), Anand, Gujarat, India.

### Experimental design and rumen sampling

Eight 5–6 year old healthy (approx. 450 kg) non-pregnant and non-lactating multiparous Kankrej cows were maintained before the start of the experiment on locally available roughages at the Livestock Research Station, Anand Agricultural University (AAU), Gujarat. Two groups of four cows were assigned to two primary diets of either dry or green roughages. Within each diet, dietary treatments were designed to have an increasing proportion of dry and green roughage and a decreasing proportion of the concentrate mix. The dietary treatments (dry/green roughage: concentrate) were K1 (50∶50); K2 (75∶25) and K3 (100∶0). The experimental animals received the K1 diet for six weeks followed by K2 for six weeks and then K3 for the subsequent six weeks. On the last day of each experimental feeding period, rumen samples were collected three hours post feeding using gastric lavage. Each rumen sample was further separated into solid and liquid fractions by squeezing through a four-layered muslin cloth and pH of the liquid fraction was measured immediately. Samples were placed on ice, transported to the laboratory and then stored at −80°C prior to analyses.

### DNA extraction

The archived rumen samples were thawed and processed separately. Solid samples were processed with PBS buffer for an hour to improve the yields of fibre adherent bacteria attached to the solid semi digested plant particles. Both solid and liquid rumen samples were then extracted for DNA using QIAamp DNA Stool Mini Kit (Qiagen, Valencia, CA). The genomic DNA was quantified and quality checked using Nanodrop (ND1000; Thermo Fisher Scientific, Wilmington, DE, USA) spectrophotometry as well as on 0.8% agarose gel electrophoresis.

### Amplification and sequencing

The choice of primers is one of the most critical steps for accurate rDNA amplicon analysis. However, there is little information available on the impact of targeting different hypervariable regions of rDNA genes to explore bacterial diversity, particularly in the rumen system. Choosing a sub-optimal or more precise primer pairs can lead to either under-representation or over-representation of particular species or even the entire phylum, and consequently leads to questionable biological conclusions [6]–[8]. Therefore, in the current study, we sought to cover the entire 16S rDNA gene using three different primer pairs and to identify the most suitable primer pair(s) that can provide a better coverage of bacterial diversity, including the rare species, in complex environments such as the rumen microbiome. The extracted DNA from both liquid and fibre rumen samples was amplified using three sets of primers (Table 1; P1: V1–V3; P2: V4–V5; P3: V6–V8) in a PCR reaction containing 5X amplification mix (5.0 µL); emPCR additive (2.0 µL); 100% DMSO (1.5 µL); 10 pM forward primer (1.0 µL); 10 pMreverse primer (1.0 µL); nuclease free water (12.5 µL); emPCR enzyme mix (1.0 µL) and 30 ng of template (1 µL). All PCR reactions were run on a thermal cycler with an initial denaturation at 95°C for 3 min followed by 35 cycles with each cycle containing denaturation at 95°C for 30 sec; annealing at 60°C for 1 min and extension at 72°C for 1 min and then a concluding step of extension at 72°C for 7 min. The amplified PCR products were size selected (+/−50 bp) using the gel cutting method, eluted using Qiaquick gel extraction kit (Qiagen, Valencia, CA) and quantified using Qubit DNA HS assay (Life Technologies, Grand Island, NY). The amplicons from the three primer pairs generated for each sample were pooled in equimolar concentration. The pyrosequencing of amplicons was performed at the OME Research Facility (Anand, Gujarat, India) using a 454 Roche Platform (GS FLX Titanium; Roche 454 Life Sciences, Branford, CT).

**Table 1:** PCR primer pair targeting different hyper variable regions of 16S rDNA.

Primer Pair Name	Primer pair	Sequence (5′-3′)	Region targeted	Amplicon length (bp)	Reference
**P1**	8F	AGA GTT TGA TCC TGG CTC AG	V1, V2 & V3	527	[73], [74]
	534R	ATT ACC GCG GCT GCT GGC			
**P2**	517F	GCC AGC AGC CGC GGT AA	V4 & V5	410	[49]
	926R	CCG TCA ATT YYT TTR AGT TT			[49]
**P3**	917F	GAA TTG ACG GGG RCC C	V6, V7 & V8	452	[49], [75]
	1386R	GCG GTG TGT GCA AGG AGC			

### Data analysis

The 16S pyrosequence reads were analyzed using the QIIME pipeline [9], followed by statistical analysis in R [10]. Reads were discarded if they did not match the expected sample-specific barcode and 16S primer sequences, shorter than 200 bp or longer than 1000 bp, or contained a homopolymer sequence in excess of 6 bp. Operational taxonomic units (OTUs) were formed at 97% similarity using UCLUST [11]. Representative sequences from each OTU were aligned to 16S reference sequences with PyNAST [12] and used to infer a phylogenetic tree with FastTree [13]. Taxonomic assignments within the GreenGenes taxonomy [12/10 release, [14] were generated using the RDP Classifier version 2.2 [15]. Alpha diversity of samples was calculated between samples of different forages, dietary treatments, rumen fractions and primer pairs at different rarefaction depths (i.e. 200, 5000 and 7000) using available preferences such as the chao1 estimator for species richness, and the Shannon diversity index, which estimates total diversity taking into account both species richness and evenness for each rarefaction depth. A non-parametric permutational multivariate ANOVA test [16], implemented in the vegan package for R [17], [18], was used to test the effects of primer pairs, dietary treatments and fraction on overall community composition, as measured by weighted UniFrac distance [19]. To test for differences in taxon abundance, a generalized non-linear model was constructed with the nlme package for R [20].

## Results

### Details of dietary composition

The nutrient and chemical composition of the two main forages along with the dietary levels (treatments) and the mean ruminal pH values in the respective dietary treatments are presented in Table 2. The dietary treatments differed in their total protein and crude fibre concentrations. DK1 and GK1 treatments contained higher protein concentrations, while DK3 and GK3 treatments had higher crude fibre concentrations. The ruminal pH for dietary treatments containing 50% concentrate (DK1 and GK1) had different pH values while K2 and K3 treatments in both dry and green roughage diets had similar pH values.

**Table 2:** Nutrient and chemical composition (%) of experimental dietary treatments and the mean ruminal pH.

Nutrient (%)	Dry	Green	Concentrate	Nutrient composition in the dietary treatments					
				DKI	DK2	DK3	GK1	GK2	GK3
**Moisture**	6.31	82.26	5.74	ND	ND	ND	ND	ND	ND
**Crude protein**	5.32	7.75	20.21	12.77	9.04	5.32	13.98	10.86	7.75
**Crude fat**	1.44	0.94	1.87	1.66	1.55	1.44	1.40	1.17	0.94
**Crude fibre**	31.00	32.85	12.57	21.79	26.39	31.00	22.71	27.78	32.85
**Acid insoluble ash**	2.53	3.36	3.84	3.19	2.86	2.55	3.6	3.48	3.36
**Ruminal pH**				7.29±0.06^c^	7.09±0.06^b^	7.08±0.05^b^	6.88±0.05^a^	6.97±0.07^ab^	6.99±0.08^ab^

DK1: 50% dry forage: 50% concentrate; DK2: 75% dry forage: 25% concentrate and DK3: 100% dry forage; GK1: 50% green forage: 50% concentrate; GK2: 75% green forage: 25% concentrate; GK3: 100% green forage; ND: Not detected;

a, b, c: means in a row having different superscript are statistically different (P<0.05).

±: standard error of means (n = 4).

### Bacterial community comparisons

**Figure 1:**
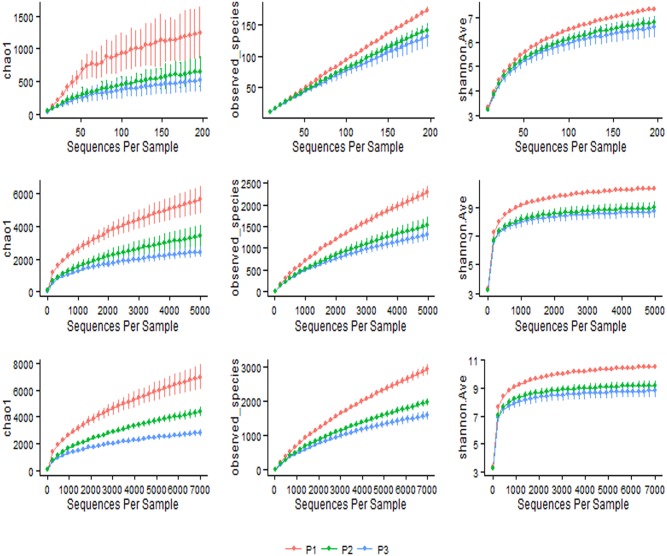
Rarefaction plots for three different primer pairs. Sequence depths a) 200, b) 5000 and c) 7000 displaying species richness (Chao 1 and Observed species) and phylogenetic relationship (Shannon index); (P1: targeting V1–V3 region; P2: targeting V4–V5 region and P3: targeting V6–V8 region).

A total of 748,700 reads from 144 different bacterial communities were analyzed in this study. Alignments and phylogenetic assignments of 16S pyrotags was performed at 97% similarity which resulted in the identification of 21 phyla and 453 genera in the bacterial domain (Table S1, S2, S3, S4). Distinct differences in species richness and diversity were evident by primer pair (Fig. 1a, b, and c). The effect of different dietary regimes is relatively small when compared to the effect of primer pairs on the distribution patterns of different bacterial species in the rumen (Fig. S1, S2 and S3).

**Figure 2:**
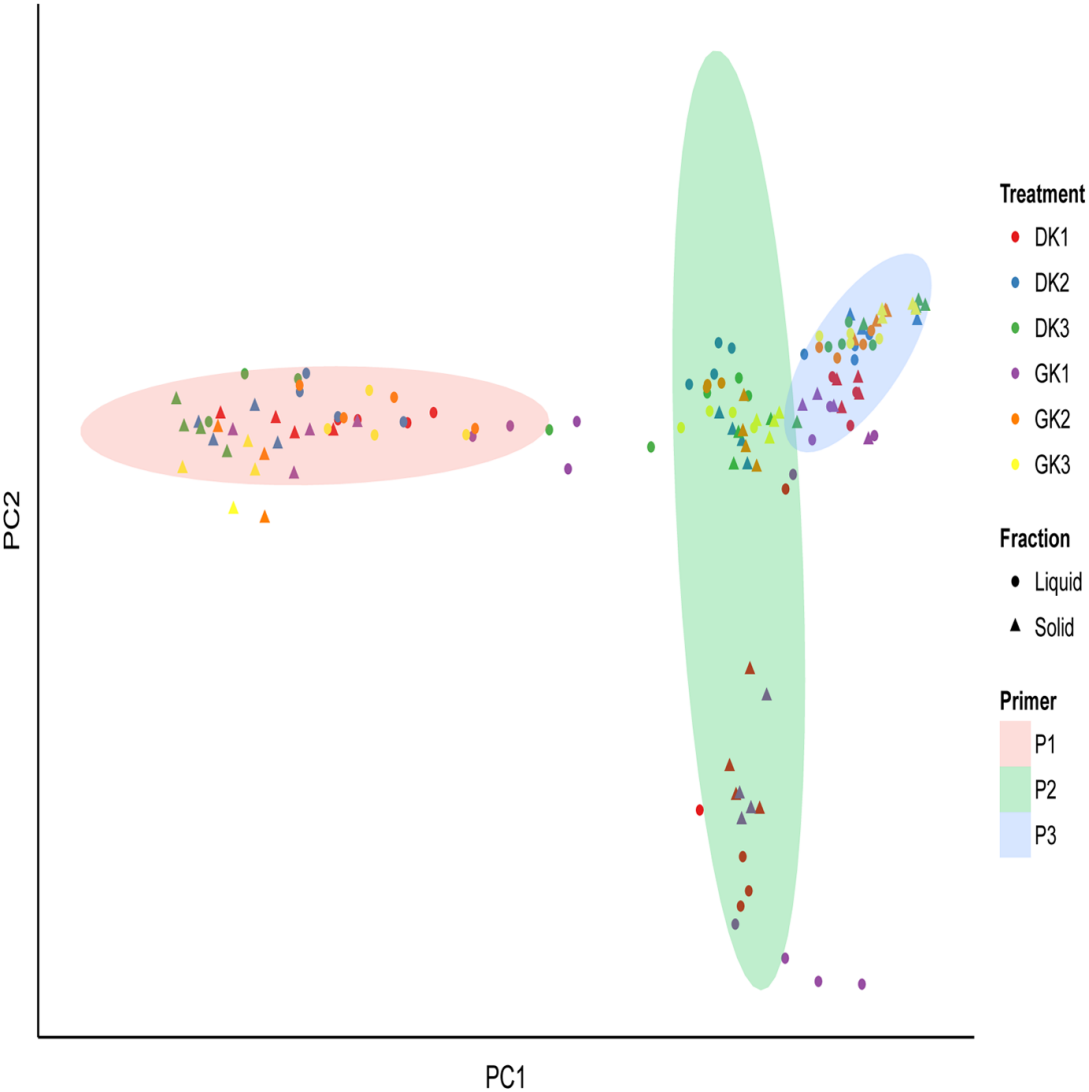
Principal coordinate analysis based on weighted Unifrac distances. Primer pair (P1: targeting V1–V3 region; P2: targeting V4–V5 region and P3: targeting V6–V8 region); treatment: (DK1: 50% dry forage: 50% concentrate; DK2: 75% dry forage: 25% concentrate and DK3: 100% dry forage; GK1: 50% green forage: 50% concentrate; GK2: 75% green forage: 25% concentrate; GK3: 100% green forage) and fraction: solid (S) and liquid (L).

Comparisons between bacterial communities were based on the UniFrac distances calculated by primer pair, dietary treatment and fraction and visualized using principle coordinate analysis (Fig. 2). Clustering of communities was influenced by the interactions between primer pair, treatment, and fraction (P<0.001; Fig. 2; Table 3). The effect of primer pair on the community composition was significant (P<0.001; Fig. 2). Bacterial community composition was influenced by dietary treatment (P<0.001) (Fig. 2; Table 3), both within and between primer pairs. It is apparent that both liquid and solid fractions had distinct community compositions (P<0.001; Fig. 2). However, there was no effect of forage (dry or green) on community composition (results not shown).

**Table 3:** Effect of dietary treatment, fraction and primer and their interactions on relative abundance of rumen bacterial phyla.

Bacterial Phyla	Individual effect			Interactions			
	P	T	F	PxT	PxF	TxF	PxTxF
***Bacteroidetes***	***	***	***	***	***	***	***
***Firmicutes***	***	***	***	***	***	**	NS
***Fibrobacteres***	***	***	***	***	***	***	***
***Proteobacteria***	***	***	***	***	NS	*	NS
***Tenericutes***	***	***	***	***	***	***	***
***Lentisphaerae***	***	***	***	***	**	***	***
***Cyanobacteria***	***	***	***	***	***	***	***
***TM7***	***	NS	**	*	***	NS	NS
***Spirochaetes***	***	***	***	NS	***	**	NS
***Verrucomicrobia***	***	***	***	***	***	***	**
***Actinobacteria***	*	**	**	-	NS	-	NS
***WPS.2***	***	***	NS	***	-	NS	NS
***Synergistetes***	NS	***	NS	NS	NS	NS	NS
***Elusimicrobia***	***	***	***	*	***	**	-
***Chloroflexi***	*	-	*	**	NS	NS	NS
***SR1***	***	*	*	***	*	NS	*
***Armatimonadetes***	***	***	**	NS	-	NS	**
***LD1***	***	NS	**	***	**	NS	NS
***X.Thermi.***	NS	***	*	NS	NS	**	NS
***Planctomycetes***	***	***	NS	***	-	**	***

P: primer; T: treatment; F: fraction; NS: Non-significant;

***: P<0.001;

**: P<0.01;

*: P<0.05.

### Phylogenetic characterization of bacterial lineages

**Figure 3:**
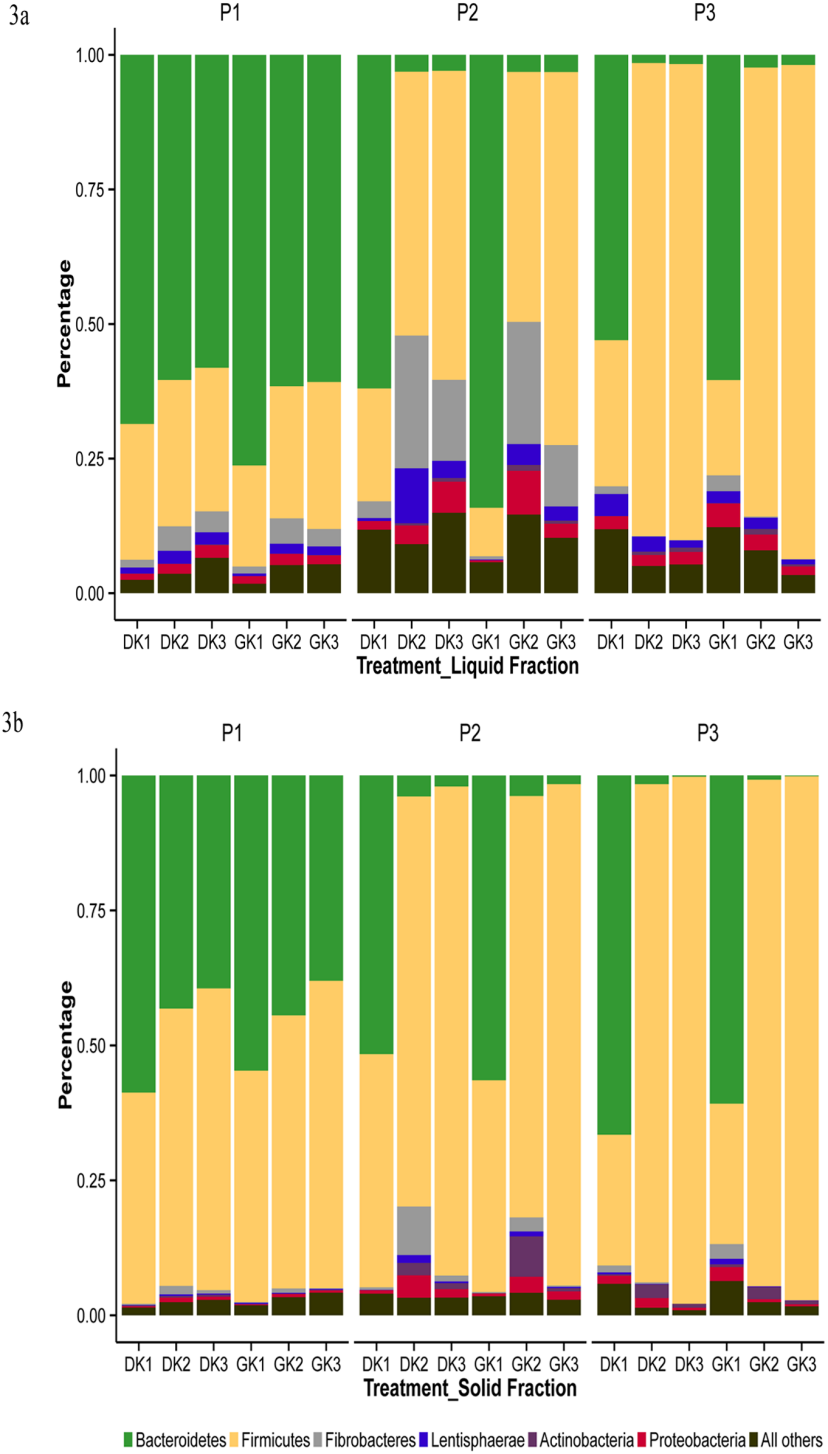
Phylogenetic composition by primer pairs and dietary treatments. Rumen fraction a) liquid; b) solid; Primer pair (P1: targeting V1–V3 region; P2: targeting V4–V5 region and P3: targeting V6–V8 region), treatment: (DK1: 50% dry forage: 50% concentrate; DK2: 75% dry forage: 25% concentrate and DK3: 100% dry forage; GK1: 50% green forage: 50% concentrate; GK2: 75% green forage: 25% concentrate; GK3: 100% green forage) and fraction: solid (S) and liquid (L).

**Figure 4:**
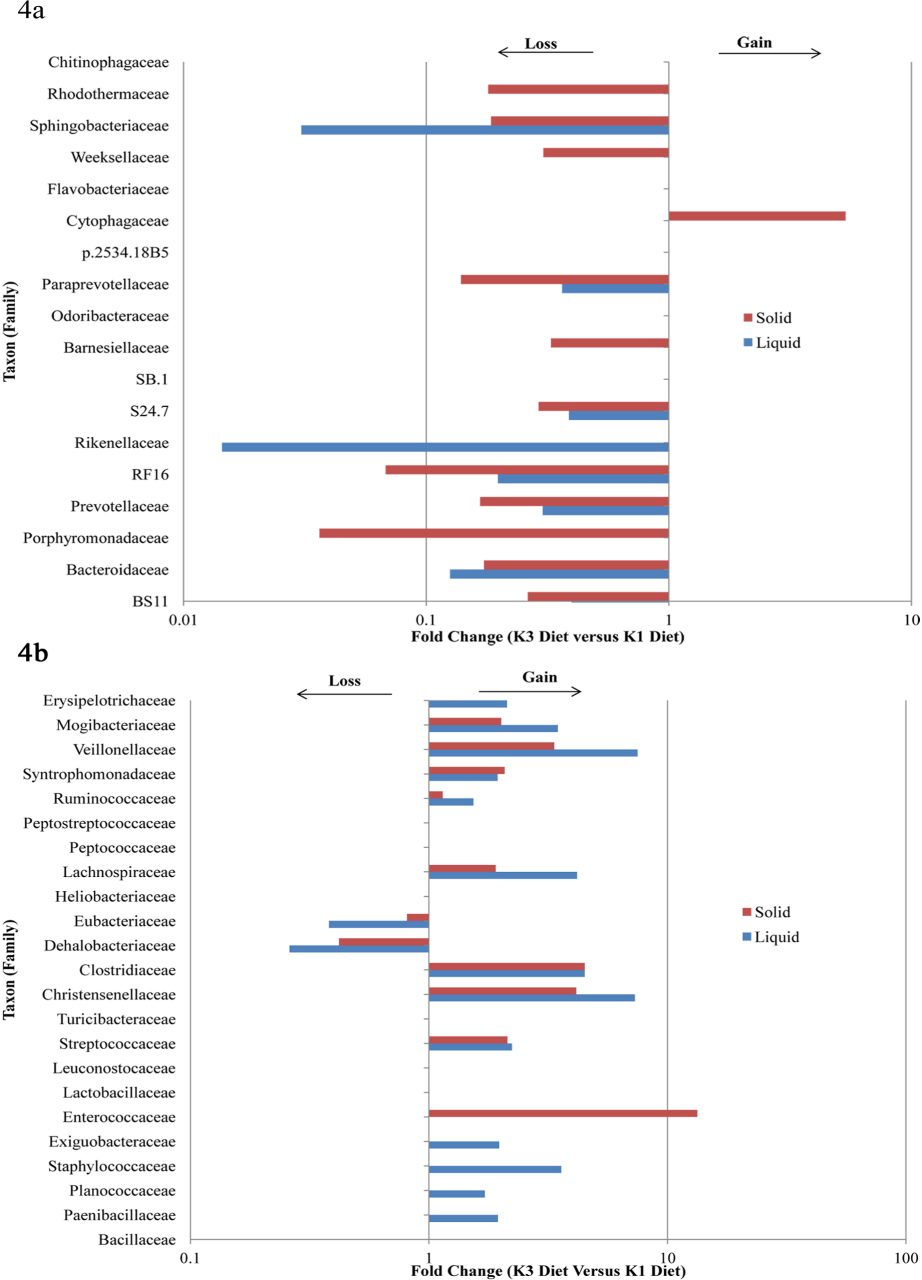
Fold changes in STabundant bacterial lineages at family level. Bacterial lineages a) loss of lineages in *Bacteroidetes*; b) gain in lineages in *Firmicutes*, across both fractions and primers, as the animals transitioned from K1 (50% dry/green forage: 50% concentrate) to K3 (100% dry/green forage).

**Figure 5:**
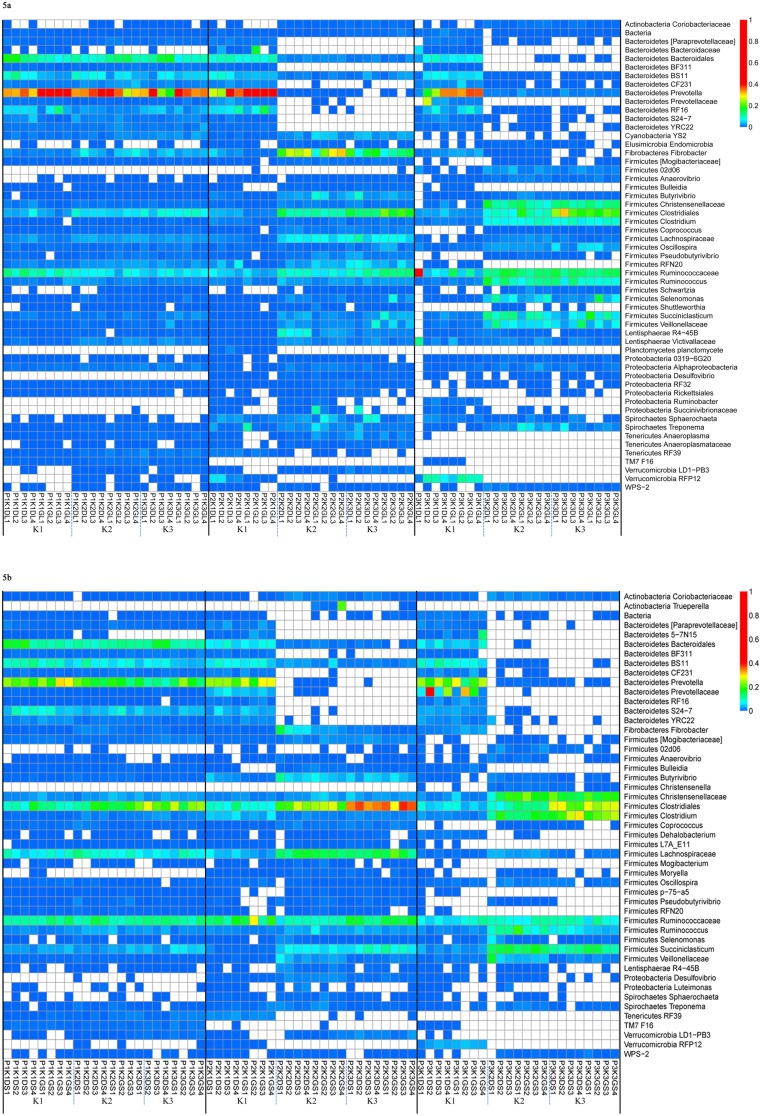
Thermal double dendrogram of the most abundant bacterial operational taxonomic units (OTUs). Rumen fraction a) liquid; b) solid; Primer pair (P1: targeting V1–V3 region; P2: targeting V4–V5 region and P3: targeting V6–V8 region), treatment: (DK1: 50% dry forage: 50% concentrate; DK2: 75% dry forage: 25% concentrate and DK3: 100% dry forage; GK1: 50% green forage: 50% concentrate; GK2: 75% green forage: 25% concentrate; GK3: 100% green forage).

Across all communities the most predominant phyla were *Bacteroidetes* and *Firmicutes* comprising up to 90% (Fig. 3a, b). We found that as the animals transitioned from K1 to K3 diets, lineages from *Bacteroidetes* reduced and that of *Firmicutes* increased in both fractions across all primer pairs (Fig. 4 a, b). Other phyla that contributed to greater than 1% abundance were *Fibrobacteres*, *Proteobacteria*, *Tenericutes*, *Lentisphaerae* and *Verrucomicrobia*. The lineages from the *Bacteroidetes* phylum were mostly assigned to the *Prevotellaceae* family. About 11 genera (including unclassified genera) were identified from the *Bacteroidetes* lineages that contributed to more than 0.2% abundance in a majority of communities (Fig. 5a, b). However, *Prevotella* was the most dominant genus across all communities. The lineages from *Firmicutes* were dominated by *Ruminococcaceae*, *Lachnospiraceae*, and *Veillonellaceae* members represented by a substantial number of genera (Fig. 5a, b).

### Shifts in the bacterial phylotypes

#### Effects of interaction

Shifts in the abundance of bacterial populations were apparent from phylum through genus (Fig. 3a, b; 4a, b; 5a, b; Table S1, S2, S3, S4). The abundance of individual bacterial populations was highly influenced by interactions between primer pair, dietary treatments and fractions (P×T×F; P×T; P×F and T×F; Table 3 and 4).

**Table 4:** Effect of dietary treatment, fraction and primer and their interactions on relative abundance of rumen bacterial taxa at the genus level.

Bacterial taxa	Individual effect			Interactions			
	P	T	F	PxT	PxF	TxF	PxTxF
***Bacteroidetes; BS11***	***	***	***	***	NS	***	NS
***Bacteroidetes; Bacteroidaceae; BF311***	***	***	***	***	NS	NS	NS
***Bacteroidetes; Porphyromonadaceae; Parabacteroides***	*	**	NS	***	NS	NS	NS
***Bacteroidetes; Prevotellaceae; Prevotella***	***	***	***	***	***	***	-
***Bacteroidetes; RF16***	*	***	***	-	***	***	NS
***Bacteroidetes; S24.7***	***	***	***	NS	***	***	NS
***Bacteroidetes; Paraprevotellaceae; CF231***	***	***	***	***	**	NS	NS
***Bacteroidetes; Paraprevotellaceae; YRC22***	***	***	***	**	-	***	-
***Bacteroidetes; Sphingobacteriaceae; Sphingobacterium***	*	***	*	***	*	***	**
***Bacteroidetes; Rhodothermaceae; Rubricoccus***	*	*	**	NS	*	*	NS
***Fibrobacteres; Fibrobacteraceae; Fibrobacter***	***	***	***	***	***	***	***
***Firmicutes; Christensenellaceae; Christensenella***	***	***	***	***	***	***	***
***Firmicutes; Clostridiaceae; 02d06***	***	***	***	***	***	NS	NS
***Firmicutes; Clostridiaceae; Clostridium***	***	***	***	***	***	***	***
***Firmicutes; Dehalobacteriaceae; Dehalobacterium***	***	***	***	***	*	*	***
***Firmicutes; Lachnospiraceae; Butyrivibrio***	***	***	**	***	*	-	NS
***Firmicutes; Lachnospiraceae; Clostridium***	**	-	-	**	NS	NS	*
***Firmicutes; Lachnospiraceae; Coprococcus***	***	***	NS	***	*	-	NS
***Firmicutes; Lachnospiraceae; Moryella***	***	***	***	*	***	NS	NS
***Firmicutes; Lachnospiraceae; Pseudobutyrivibrio***	***	***	***	**	***	***	NS
***Firmicutes; Lachnospiraceae; Syntrophococcus***	***	-	**	*	***	**	*
***Firmicutes; Ruminococcaceae; Oscillospira***	***	***	***	**	***	***	NS
***Firmicutes; Ruminococcaceae; Ruminococcus***	***	***	NS	***	*	*	NS
***Firmicutes; Veillonellaceae; Anaerovibrio***	***	***	*	***	***	***	NS
***Firmicutes; Veillonellaceae; Mitsuokella***	**	**	**	***	*	*	**
***Firmicutes; Veillonellaceae; Schwartzia***	***	***	***	***	***	***	***
***Firmicutes; Veillonellaceae; Selenomonas***	***	***	***	***	***	***	-
***Firmicutes; Veillonellaceae; Succiniclasticum***	***	***	***	***	NS	***	-
***Lentisphaerae; R4.45B***	***	***	***	***	***	***	***
***Proteobacteria; Caulobacteraceae; Mycoplana***	***	***	**	***	***	***	***
***Proteobacteria; Desulfovibrionaceae; Desulfovibrio***	***	***	***	***	***	***	***
***Proteobacteria; Succinivibrionaceae; Ruminobacter***	***	***	*	***	*	*	NS
***Proteobacteria; Succinivibrionaceae; Succinivibrio***	***	***	***	***	***	***	***
***Tenericutes; Anaeroplasmataceae; Anaeroplasma***	***	***	***	**	***	***	**
***Verrucomicrobia; RFP12***	***	***	***	***	***	***	***

P: primer; T: treatment; F: fraction; NS: Non-significant;

***: P<0.001;

**: P<0.01;

*: P<0.05.

Among the bacterial phyla, *Bacteriodetes*, *Fibrobacteres*, *and Tenericutes were* greatly influenced (P<0.001) by P×T×F; P×T; P×F and T×F interactions (Table 3; Tables S1, S2). Among the *Bacteroidetes* representatives, *Prevotellaceae* (*Prevotella*, *YRC22*) and *Sphingobacteriaceae* (*Sphingobacterium*) were influenced by nearly all interactions. Similarly the clans of *Firmicutes* such as *Clostridia* (*Clostridium*, *Cristensella*, *Dehalobacterium*), *Lachnospiraceae* (*Butyrivibrio*, *Syntrophococcus*, *Psuedobutyrivibrio*), *Ruminococcaceae* (*Oscillospira*, *Ruminococcus*) and *Veillonellaceae* (*Schwartzia*, *Selenomonas*, *Succiniclasticum*) changed due to interactions. Genus *Fibrobacter* of the phylum *Fibrobacteres* and the *Desulfovibrio* and *Succinivibrio* lineages of *Proteobacteria* were also significantly influenced by the interactions among primer, fraction and treatment (Table 4; Tables S3, S4).

### Distinction between community profiles of the fibre and liquid fraction

Although the same bacterial lineages were commonly present in fibre and liquid fractions, their percent abundance varied (P<0.001) between the two fractions (Fig. 3a, b; Fig. 5a, b).

In the liquid fraction, among communities associated with primer pair 1, the predominant phylum was *Bacteroidetes* (up to >70%). The proportion of *Bacteroidetes* was altered with changes in dietary treatments with K1 showing higher abundance of *Bacteroidetes* while the abundance was reduced from K1 to K3 (P<0.001). Green roughage fed animals had a slightly higher abundance of *Bacteroidetes* than dry roughage fed animals. Although the contribution from *Firmicutes* was substantial, there was little differentiation between the K1, K2 and K3 treatments. A higher abundance of *Proteobacteria* and *Fibrobacter* was noticed in the K2 treatment (P<0.001).

Across bacterial communities recovered from primer pair 2 in the liquid fraction, the dominant phylum was dependent on the dietary treatment. The K1 treatment had a significant (P<0.001) abundance of *Bacteroidetes* (60–80%) which reduced to 3.0% in the K2 and K3 treatments. In contrast, *Firmicutes* was 20% in the K1 treatments which significantly (P<0.001) increased to 60% abundance in the K3 treatment. The abundance of *Proteobacteria* and *Fibrobacter* was also substantial in the K2 and K3 treatments compared to the K1 treatment.

Primer pair 3 derived bacterial communities showed a similar pattern to that of primer pair 2 in the liquid fraction. However, the abundance of *Firmicutes* was much higher (about 90%) in the K2 and K3 treatments. Also the recovery of *Proteobacteria* and *Fibrobacteres* was lower with primer pair 3 as compared to other primer pairs.

In the solid fraction, P1 associated bacterial communities showed lower abundance values for *Bacteroidetes* and higher values for *Firmicutes* compared to the liquid fraction. Across P2 communities, K1 treatment had a comparable profile to that of K1 in P1 associated communities. However, K3 treatments were dominated by *Firmicutes* (up to 75%). In K2 treatments, the contribution from *Firmicutes* was up to 55% while *Proteobacteria*, *Fibrobacteres* and *Actinobacteria* together contributed up to 45%. The abundance of *Bacteroidetes* in K2 and K3 regimen was minimal (1–3%). In P3 associated communities, the abundance of *Bacteroidetes* and *Firmicutes* was 60∶20 in the K1 diets. However, on K2 and K3 dietary treatments, *Firmicutes* alone comprised more than 95% abundance.

### Comparison of bacterial fingerprints at the lowest level of lineage

We chose to present the abundance (>0.2%) of bacterial lineages at the OTU level for each of the samples in both fractions (Fig. 5a and b). For ease of interpretation, fingerprints were presented by primer pair, dietary treatments and fractions. The effect of interactions between primer pair, treatment, and fraction on the abundance of bacterial genera is presented (Table 4).

In the liquid fraction, about 55 lineages were identified with 11 lineages from *Bacteroidetes* and 20 lineages from *Firmicutes* across all samples. In P1 associated communities, genus *Prevotella* was well represented along with several other *Bacteroidetes* lineages. The majority (about 19 out of 20) of lineages from *Firmicutes* except for *Clostridiaceae* (02d06) were recovered by P1. However, *Ruminobacter*, *Desulfovibrio* and *Succinovibrionaceae members were* not recovered; representatives from *Verrucomicrobia*, and *Elusimicrobia* were weakly represented across P1 associated communities. In P2 associated profiles, K1 had contrasting profiles compared to K2 and K3. Notably, except for the weak presence of *Prevotella*, all other lineages of *Bacteroidetes* were not detected in K2 and K3 treatments. On the contrary, diversity in *Firmicutes* was high with more representative OTUs present in all P2 associated communities; however, their abundance was much higher in K2 and K3 treatment profiles. Also, the abundance of *Fibrobacteres* was much more evident in K2 followed by K3 communities. The P3 associated communities showed different profiles compared to P1 and P2 primer pairs. Among the *Bacteroidetes* lineages, lineages from *Prevotellaceae* were only detected in K1 communities. The abundance of *Prevotella* was much higher in K1 compared to K2 and K3. The representatives from *Christensenellaceae*, *Ruminococcaceae*, *Clostridiales* and *Vellionellaceae* were abundant on K2 and K3 treatments among P3 communities. The OTUs from *Planctomycete* and *Tenericutes* were not recovered by primer pair 3.

In the solid fraction, 49% lineages were identified from *Firmicutes* and 22% from *Bacteroidetes*. In P1 associated communities, all the lineages from *Bacteroidetes* and *Firmicutes* were well represented in K1, K2 and K3 dietary treatments except *Firmicutes* 02d06. However, members of *Proteobacteria* (*Desulfovibrio*, *Luteimonas*) and *Verrucomicrobia* (RFP12) were either weakly recovered or not detected in K1, K2 and K3 treatments. In P2 associated patterns, lineages from *Bacteroidetes* were more abundant in K1 compared to K2 and K3, where most of the lineages were not detected. Among *Firmicutes*, *Clostridium* followed by *Lachnospiraceae*, *Succiniclasticum* and *Butyrivibrio* were more abundant in K2 and K3 whereas *Ruminococcaceae* was abundant in K1 treatment. Primer pair 2 showed higher abundance of *Fibrobacter* in K2 and K3 compared to P1 and P3. The OTUs from *Verrucomicrobia* RFP12 and *Tenericutes* RF39 were not detected in K2 and K3 with P2 whereas TM7 F16 was identified in all the treatments.

In P3 associated microbial profiles, results for *Bacteroidetes* lineage were similar to that of P2 in K1 diet whereas in K2 and K3 more *Bacteroidetes* lineages were recovered by P3 compared to P2. The abundance of *Christensenellaceae*, *Clostridiales*, *Clostridium*, *Ruminococcus*, *Succiniclasticum* and *Veillonellaceae* were much higher in K2 and K3, whereas *Bulleidia* and *Firmicutes* RFN20 were not detected in these diets. The members of *Proteobacteria* (*Ruminobacter* and *Succinivibrionaceae*) were not detected in K2 and K3 compared to K1. The OTUs from *Anareoplasmataceae*, *Firmicutes* RF39, and *Verrucomicrobia* LD1-PB3 were not recovered by primer pair 3.

## Discussion

The concept of the “microbiome” (microbes, their genes and interactions with the host/habitat) is currently being evaluated in many aspects of biological science, and studies over the past decade have been dramatically advanced by Next Generation Sequencing (NGS) technology [21], [22]. For example, characterization of the rumen microbiome and its associated repertoire of glycoside hydroxylase (GH) enzymes in steers using NGS revealed that the microbiome composition, including GH content, is driven primarily by diet [23].

Our study intends to characterize the rumen microbiome of Kankrej cattle, an indigenous bovine breed of the Indian subcontinent which is commonly reared to serve multiple needs such as milk, meat and draft purposes. The aim of this study is to understand the rumen microbiome of this indigenous breed and also elucidate the dynamics in the rumen microbial communities mediated by a difference in primer pairs, fractions and dietary treatments in the rumen contents using 16S rDNA pyrotag sequencing technology.

Bacterial populations within the rumen microbiome have been categorized into three major groups based on their location designated as adherent bacteria (bound to feed particles), planktonic bacteria (free-living in the liquid) and the epimural community (associated with rumen epithelium) [24], [25]. Previously, either whole rumen contents or the squeezed rumen fluid was used for bacterial diversity analysis; however, differentiating microbial communities by rumen fraction has recently become more common due to the efficiency and lower cost of NGS technology [5], [23]–[29]. Similar to our findings distinct microbial communities associated with each of the rumen fractions have been observed across several reports [5], [29], [30]. Further, we found a contrasting difference in the phylogenetic composition of each fraction at the phylum level with a higher abundance of *Bacteroidetes* in the liquid fraction similar to findings of [5] and a higher abundance of *Firmicutes* in the solid fraction analogous to the reports of [21], [31]. *Firmicutes* lineages are known to utilize readily available fermentable carbohydrates [31] and also participate in the initial colonization of the peripheral side chains of cellulosic matrix [23] thus showing their metabolic role in carbohydrate digestion. In contrast, *Bacteroidetes lineages* are reported to have diverse metabolic capabilities including the degradation of protein and polysaccharides [31], [32].

A majority of studies rely on 16S rRNA gene to understand the phylogenetic composition of bacterial communities utilizing either cultivation [33], [34] or cultivation independent DNA derived next generation sequencing technology [5], [35]–[37]. However, amplification of 16S rDNA gene fragments can be biased owing to differences in primer pairs used to target different hyper-variable regions of the 16S rDNA gene [5], [38]–[41]. In addition, differences in sampling procedure used to harvest rumen contents (gastric tube vs. cannulated animal) and sample type (whole rumen contents vs. separate rumen fractions) can have a huge impact on microbial diversity [42]–[44]. Considering the above factors that account for variation, and for the fact that we have identified a strong influence of primer pair on the recovery of bacterial populations in the rumen of water buffalo [5], we investigated the influence of different primer pairs on the rumen microbiome of Kankrej cattle.

Congruent to our earlier report [5], we found that bacterial diversity was contingent upon the choice of primer pairs. However, we have identified that the effect of interactions between primer pair, dietary treatment and fraction had a strong influence on the community composition as well as upon the abundance of individual bacterial lineages. In agreement with previous studies [5], [23], [29], *Bacteroidetes* and *Firmicutes* were found to comprise about 90% of the bacterial populations regardless of the difference in dietary treatment, primer and fraction. Within each primer pair, the effect of dietary treatments was more pronounced on the abundance of either *Bacteroidetes* or *Firmicutes* in P2 and P3 associated communities while both phyla were co-dominant in P1 associated communities. Our previous report [5] showed a greater recovery of *Bacteroidetes* with the P2 primer pair in contrast to this study. Differences in the recovery of *Bacteroidetes* between Pitta et al [5] and the current study is largely explained by the amplification of cDNA from metabolically active bacteria in our previous work compared to total bacterial DNA (live and dead) in the present study. In addition, differences in the host animal (water buffalo vs Kankrej cattle) could also be a confounding factor in bacterial diversity determination. Previous reports [5], [45]–[47] have also demonstrated that the bacterial diversity is host specific. Future work should aim to investigate bacterial diversity based upon both DNA-derived and cDNA-derived 16S rDNA amplicons from a single species of ruminants to study the influence of primer pair on total and metabolically active bacterial diversity.

Notably, primer pair P2 was able to retrieve lineages of *Proteobacteria*, *Fibrobacteres*, *Tenericutes* and *Spirochaetes* much more efficiently at the expense of *Bacteroidetes* particularly in the liquid portion than the other two primer pairs possibly due to an interaction effect of dietary treatment, fraction and primer pair. From these results it is apparent that the detection and/or recovery of certain phyla are primer dependent. This primer effect has been reported in other microbial ecosystems. The effect of seven different primer pairs, targeting different hypervariable regions of DNA derived from activated sludge [38] and marine samples [6] demonstrated that combining V3 and V4 regions yielded better diversity patterns. It was also reported that V3–V4 and V4–V5 hyper variable regions were recommended for optimal bacterial profiling based on *in* *silico* analysis [38], [48]–[50]. Across different ecosystems including the rumen microbiome, the use of primer pairs that flank V3–V4 hypervariable regions resulted in the recovery of a majority of bacterial populations [5], [41], [49]–[53] which partially concurs with this study. However, Claesson et al. [48] revealed significant amplification bias with the experimental sequencing of the V3–V4 region compared to the other regions, accentuating the necessity for more experimental validation of primer pairs. Based on our results, P1 primer pair, targeting V1–V3 hyper-variable region, was found to offer the more informative fingerprinting profiles with the rumen fractions (solid and liquid) as well as with the dietary treatments compared to P2 and P3 primer pairs. Yu and Morrison [54] also suggested amplification of V1 and V3 region for gut microbiome studies with short amplicon size, and thus corroborate well with our study.

Diet has a direct influence on the composition of the rumen microbiome [4], [34] and studies have elucidated diet-induced shifts in the microbiome using different molecular techniques [5], [26], [29], [52], [53], [55], [56]. In our study, animals had access to either dry or green roughage in increasing proportion while the proportion of concentrate declined as the animals moved from K1 to K3 diets. As the ratio between the concentrate and roughage changed, a corresponding change was noticed in the phylogenetic composition of rumen bacterial populations in Kankrej cattle. Moreover, difference in phylogenetic composition was also noticed due to different primer pairs.

Similar to previous findings [5], [55], we have noted a higher abundance of *Bacteroidetes* with increasing proportion of concentrate in the dietary treatment with P1 primer in both solid and liquid fractions compared to P2 and P3. Coverage of *Prevotella* and other *Bacteroidetes* lineages was greater with P1 primer pair covering the V1–V3 region. The abundance of lineages of *Firmicutes* (*Coprococcus*, *Lactonifactor*, *Sporobacter*) and *Fibrobacteres* (*Fibrobacter*) was also well represented with P1 and P2 primer pairs compared to P3 whereas TM7 was not recovered with P2. Although the percent abundance was different due to the dietary effects in both solid and liquid fractions, the P1 primer pair offered more information on different bacterial phyla and covered more diverse bacterial lineages. The coverage of observed number of sequences, diversity and species richness with V1–V3 based primer (P1) was higher and in agreement with the results of [27], [57]. The present data also relates with our transition cow study which showed better coverage of bacterial lineages using V1–V2 region based primer [58]. Results from metagenomic studies (unpublished data) are also in agreement which showed more than 80% abundance of *Bacteroidetes* and *Firmicutes* in the rumen microbiome followed by *Proteobacteria*. It was reported that the main functional role of *Bacteroidetes* is polysaccharide degradation [32]. However, lineages of *Bacteroidetes* are plastic as they continue to evolve and adapt to dietary substrates that become available in the host. Therefore, *Bacteroidetes* complement host metabolism and develop the repertoire of enzymes that can target polysaccharides such as cellulose, pectin and xylan [32], oligosaccharides [59], and also host derived carbohydrates such as mucin and chondroitin sulfates containing N-glycans [60]. Comparative analysis of *Bacteroidetes* genomes revealed that these lineages contain numerous carbohydrate enzymes that can degrade different substrates originated from plant, algae and fungi due to the presence of Polysaccharide Utilizing Loci that help ligate and uptake of carbohydrate substrate and TonB receptors that transport these complexes into the cytoplasm [32]. In addition, certain lineages of *Bacteroidetes* such as *P. ruminocola* [61] and *P. albensis* [62] found in the rumen are inclined towards utilizing the dipeptides due to the presence of dipeptidyl peptidases. The abundance of *Prevotellaceae* and *Porphyromonadaceae* among the *Bacteroidetes* on concentrate diets as observed in this study is congruent with previous reports [5], [55], [63], [64]. It could be inferred that our findings corroborate with Thomas et al [32] on the diverse nature of *Bacteroidetes* and its participation in the degradation of protein and polysaccharides, both of which are available in the K1 dietary treatments.

*Firmicutes* are primarily comprised of Gram positive, low G+C content bacteria [65] and include a majority of the fibre-adherent rumen bacterial populations [23] and also in animals fed high forage diets [55]. In our study, *Firmicutes* constituted the majority of bacterial populations particularly in the solid fraction on K2 and K3 dietary treatments which were rich in crude fibre content. Among the *Firmicutes* group, families such as *Lachnospiraceae*, *Ruminococcaceae*, *Veillonellaceae* and unclassified *Clostridiales* were abundant in our study similar to previous findings [44], [57], [66]. Several reports emphasize the role of *Firmicutes*, in particular members of *Clostridiales*, *Ruminococcaceae* and *Lachnospiraceae* in fibre digestion [67]–[72] and therefore their abundance on K2 and K3 diets in the rumen of Kankrej cattle was expected.

While the abundance of *Bacteroidetes* and *Firmicutes*, including their possible metabolic capacities were evident, the shifts noted in other minor groups such as *Proteobacteria*, *Fibrobacteres*, *Verrucomicrobia*, *Spirochaetes* and unclassified bacteria that were detected in the present study remain obscure. However, their abundance was driven by interactions between primer pairs, dietary treatment and fractions. This study illustrates that the rumen microbiome of Kankrej cattle is sensitive to changes in the diet and that distinct microbial communities were identified in each of the rumen fractions. The recovery of rumen bacterial populations was dependent on primer choice, however, community and individual bacterial populations within a primer pair were driven by an interaction effect. Further, a period of six weeks was found to be adequate to identify differences in the bacterial communities due to a change in the diet. Prolonged feeding of these experimental diets could have led to the identification of a core microbial consortium that is specific for either concentrate or fibre rich diets. It is evident that the rumen microbiome of Kankrej cattle is sensitive to external stimuli and therefore further investigations can lead to novel insights to the microbial ecology as well as biotechnology research in biofuel production.

## Supporting Information

Figure S1 **Rarefaction plots for two different forages.** Sequence depths a) 200, b) 5000 and c) 7000 displaying species richness (Chao 1 and Observed species) and phylogenetic relationship (Shannon index); (D: dry and G: green).
(TIF)

Figure S2 **Rarefaction plots for six different dietary treatments.** Sequence depths a) 200, b) 5000 and c) 7000 displaying species richness (Chao 1 and Observed species) and phylogenetic relationship (Shannon index); **(**DK1: 50% dry forage: 50% concentrate; DK2: 75% dry forage: 25% concentrate and DK3: 100% dry forage; GK1: 50% green forage: 50% concentrate; GK2: 75% green forage: 25% concentrate; GK3: 100% green forage**).**
(TIF)

Figure S3 **Rarefaction plots for two different fractions.** Sequence depths a) 200, b) 5000 and c) 7000 displaying species richness (Chao 1 and Observed species) and phylogenetic relationship (Shannon index); (S: solid and L: liquid).
(TIF)

Table S1 **Mean values of bacterial phyla in the liquid fraction presented for each dietary treatment retrieved by each primer pair in the rumen of Kankrej cattle.** Primer pair (P1: targeting V1–V3 region; P2: targeting V4–V5 region and P3: targeting V6–V8 region), treatment: (DK1: 50% dry forage: 50% concentrate; DK2: 75% dry forage: 25% concentrate and DK3: 100% dry forage; GK1: 50% green forage: 50% concentrate; GK2: 75% green forage: 25% concentrate; GK3: 100% green forage).
(XLSX)

Table S2 **Mean values of bacterial phyla in the solid fraction presented for each dietary treatment retrieved by each primer pair in the rumen of Kankrej cattle.** Primer pair (P1: targeting V1–V3 region; P2: targeting V4–V5 region and P3: targeting V6–V8 region), treatment: (DK1: 50% dry forage: 50% concentrate; DK2: 75% dry forage: 25% concentrate and DK3: 100% dry forage; GK1: 50% green forage: 50% concentrate; GK2: 75% green forage: 25% concentrate; GK3: 100% green forage).
(XLSX)

Table S3 **Mean values of bacterial genus in the liquid fraction presented for each dietary treatment retrieved by each primer pair in the rumen of Kankrej cattle.** Primer pair (P1: targeting V1–V3 region; P2: targeting V4–V5 region and P3: targeting V6–V8 region), treatment: (DK1: 50% dry forage: 50% concentrate; DK2: 75% dry forage: 25% concentrate and DK3: 100% dry forage; GK1: 50% green forage: 50% concentrate; GK2: 75% green forage: 25% concentrate; GK3: 100% green forage).
(XLSX)

Table S4 **Mean values of bacterial phyla in the solid fraction presented for each dietary treatment retrieved by each primer pair in the rumen of Kankrej cattle.** Primer pair (P1: targeting V1–V3 region; P2: targeting V4–V5 region and P3: targeting V6–V8 region), treatment: (DK1: 50% dry forage: 50% concentrate; DK2: 75% dry forage: 25% concentrate and DK3: 100% dry forage; GK1: 50% green forage: 50% concentrate; GK2: 75% green forage: 25% concentrate; GK3: 100% green forage).
(XLSX)
