# Supplementary figures and images for: Bacterial Diversity Dynamics Associated with Different Diets and Different Primer Pairs in the Rumen of Kankrej Cattle
Source: PLoS One. 2014 Nov 3;9(11):e111710. doi: 10.1371/journal.pone.0111710 (PMC4218807; doi:10.1371/journal.pone.0111710)

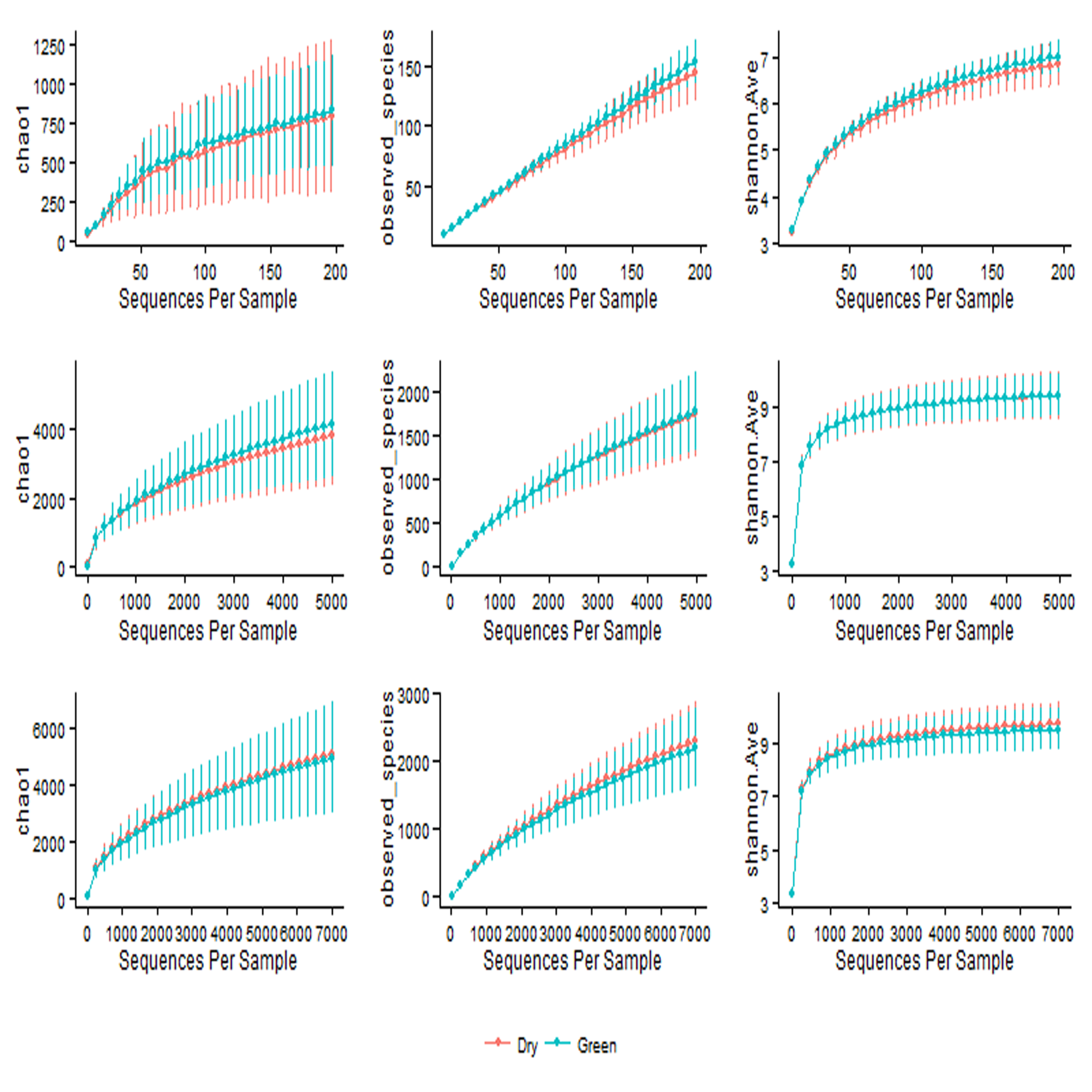

Supplement: Figure S1 — Rarefaction plots for two different forages. Sequence depths a) 200, b) 5000 and c) 7000 displaying species richness (Chao 1 and Observed species) and phylogenetic relationship (Shannon index); (D: dry and G: green). (TIF) [file pone.0111710.s001.tif]

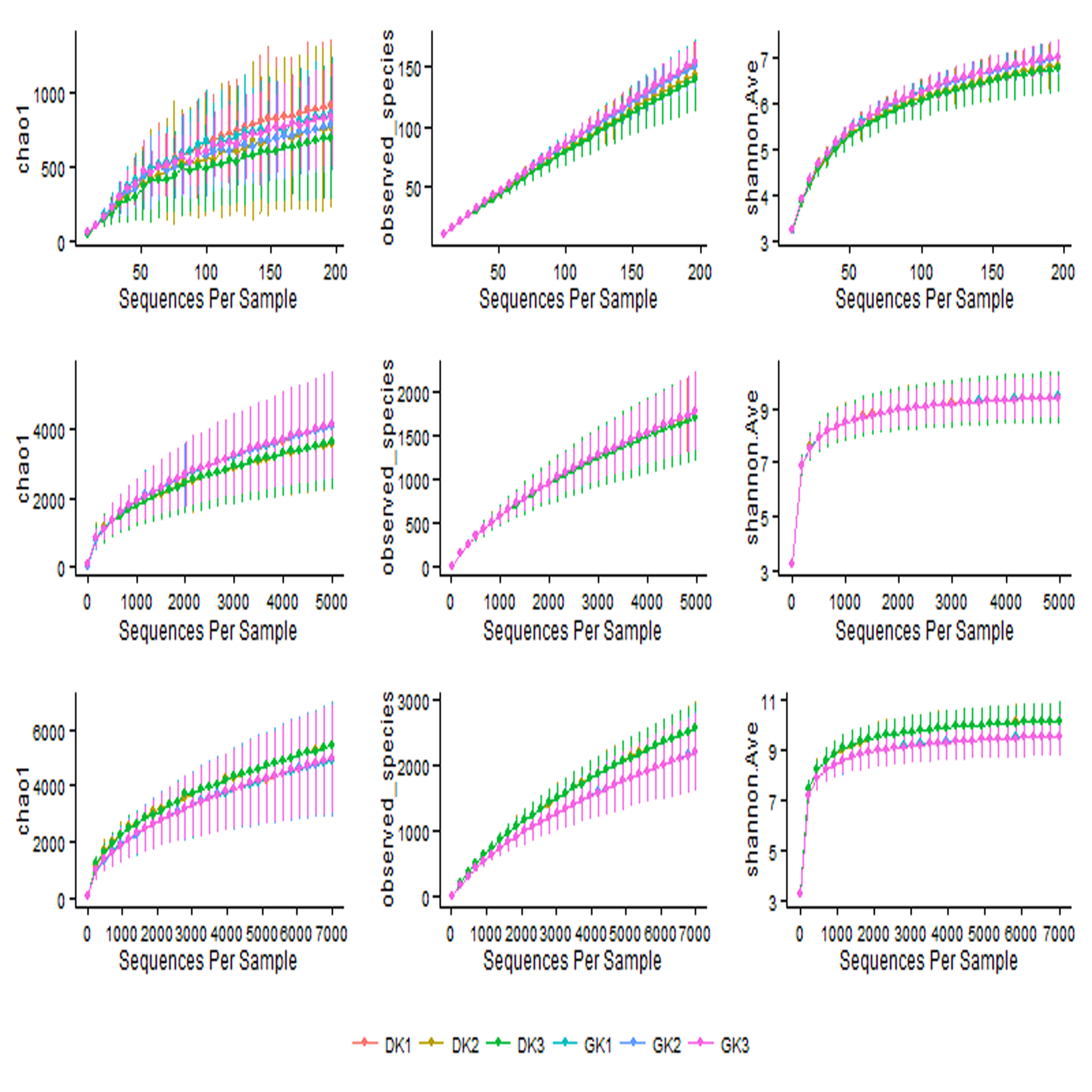

Supplement: Figure S2 — Rarefaction plots for six different dietary treatments. Sequence depths a) 200, b) 5000 and c) 7000 displaying species richness (Chao 1 and Observed species) and phylogenetic relationship (Shannon index); (DK1: 50% dry forage: 50% concentrate; DK2: 75% dry forage: 25% concentrate and DK3: 100% dry forage; GK1: 50% green forage: 50% concentrate; GK2: 75% green forage: 25% concentrate; GK3: 100% green forage). (TIF) [file pone.0111710.s002.tif]

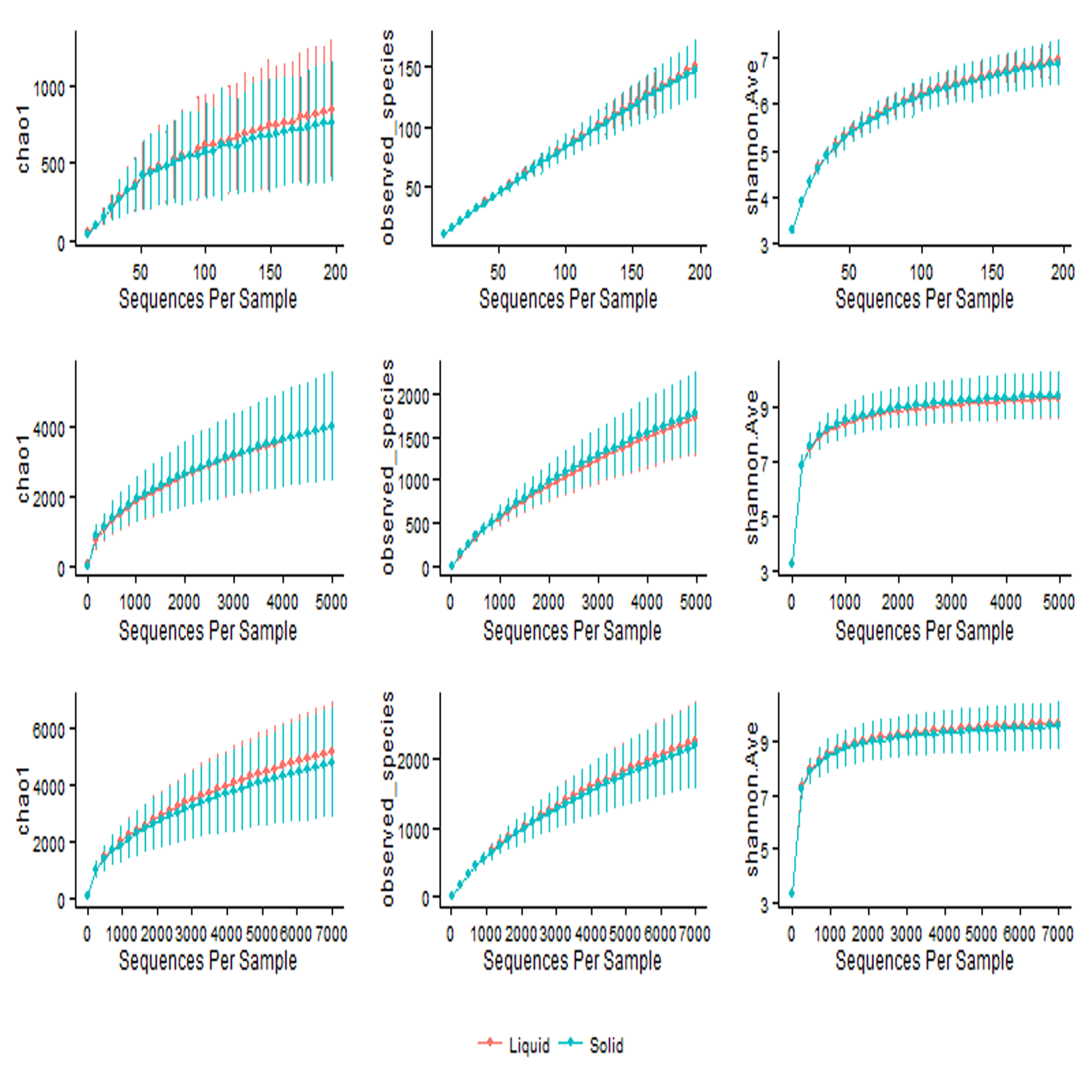

Supplement: Figure S3 — Rarefaction plots for two different fractions. Sequence depths a) 200, b) 5000 and c) 7000 displaying species richness (Chao 1 and Observed species) and phylogenetic relationship (Shannon index); (S: solid and L: liquid). (TIF) [file pone.0111710.s003.tif]
